# Equity in Modifying Plaque of Women With Undertreated Calcified Coronary Artery Disease: Design and Rationale of EMPOWER CAD study

**DOI:** 10.1016/j.jscai.2024.102289

**Published:** 2024-10-28

**Authors:** Margaret McEntegart, Nieves Gonzalo, Lahn Fendelander, Nick E.J. West, Alexandra J. Lansky

**Affiliations:** a Center for Interventional Cardiovascular Care, NewYork-Presbyterian Hospital/Columbia University Irving Medical Center, New York, New York; b Interventional Cardiology Department, Clinico San Carlos University Hospital, Madrid, Spain; c Shockwave Medical, Santa Clara, California; d Division of Cardiovascular Medicine, Department of Internal Medicine, Yale School of Medicine, New Haven, Connecticut

**Keywords:** arteriosclerosis, cardiovascular disease, coronary artery disease, intravascular lithotripsy, percutaneous coronary intervention

## Abstract

**Background:**

Coronary artery disease (CAD) is the leading cause of death for women, yet they remain underrepresented in interventional CAD studies. Women have been shown to be at increased risk of mortality and major adverse events after percutaneous coronary intervention (PCI). The poorer outcomes are likely because women are typically diagnosed with CAD late, at an older age, with more comorbidities, and with more challenging anatomy including smaller vessels and higher prevalence of coronary artery calcification.

**Methods:**

The EMPOWER CAD study (NCT05755711) is a postmarket, prospective, multicenter, single-arm observational study of the Shockwave Coronary intravascular lithotripsy (IVL) system for the treatment of women with calcified coronary artery disease. The study will enroll 400 female patients referred for PCI with coronary IVL and stenting. The primary safety end point is target lesion failure (TLF) at 30 days, defined as a composite of cardiac death, target vessel myocardial infarction, or ischemia-driven target lesion revascularization. The primary effectiveness end point is procedural success, defined as stent delivery with a residual in-stent stenosis ≤30% in all target lesions and without in-hospital TLF as assessed by an independent core laboratory and clinical events committee. Patients will be followed up for 3 years.

**Conclusions:**

The EMPOWER CAD study will enroll real-world female patients. Adjunctive use of IVL with other calcium modification technologies will be assessed, as well as a subcohort analysis of patients with optical coherence tomography imaging. The EMPOWER CAD study therefore directly addresses the underrepresentation of women in interventional cardiology clinical trials.

## Introduction

Coronary artery disease (CAD) is the leading cause of death worldwide,^1^^,^^2^ and percutaneous coronary intervention (PCI) has become the most frequently-used treatment strategy for both stable and unstable coronary syndromes.^3^, ^4^, ^5^ Further investigations of the effect of sex on PCI outcomes are necessary as women are consistently underrepresented in clinical trials,^6^, ^7^, ^8^, ^9^ and while women and men share many of the usual CAD risk factors such as age, hypertension, and diabetes, women also have other unique risk factors including pregnancy-related disorders or menopause.^10^ Additionally, compared with men, women are typically diagnosed with CAD at an older age, with more comorbidities, and with different etiologies such as nonobstructive CAD.^11^, ^12^, ^13^ As a result, women have increased mortality, major adverse cardiovascular events (MACE), myocardial infarction (MI), and target lesion revascularization after PCI.^9^^,^^11^^,^^14^

Calcified lesions remain one of the principal predictors of poor outcomes after PCI, as calcified plaque can limit delivery and expansion of balloons and stents.^15^^,^^16^ Severe calcification is associated with early adverse events such as bleeding or dissection^6^ as well as late events including restenosis, stent thrombosis, and target vessel revascularization.^17^, ^18^, ^19^ Women have smaller vessel diameters, less necrotic core volumes, higher plaque density, and higher coronary artery calcium scores, all further increasing their risk of poor outcomes.^7^^,^^20^

There are several calcium modification technologies available, but their safety appears to be different in women than that in men. Women have been reported to have increased rates of dissection, tamponade, and bleeding after rotational atherectomy compared with men.^21^^,^^22^ While the European Multicenter Euro4C Registry did not find significant bleeding differences after rotational atherectomy, women had worse in-hospital and 1-year MACE rates.^23^ Both men and women had low rates of death, MI, and target vessel revascularization after orbital atherectomy^15^^,^^24^; however, women had significantly higher rate of severe dissection.^15^ The Shockwave intravascular lithotripsy (IVL) system (Shockwave Medical) has lithotripsy emitters mounted on a traditional catheter platform that deliver localized sonic pressure waves to modify vascular calcium.^25^ In the Disrupt CAD III and IV studies, low and equivalent rates of serious angiographic complications and MACE were seen in both men and women at 1 year.^16^^,^^26^

With the challenges presented by calcified lesions, a recent expert consensus identified the need for further sex-specific studies on the safety and effectiveness of calcified plaque modification strategies.^1^ The EMPOWER CAD study (NCT05755711) design allows for all-comer female patients, including those with acute coronary syndromes and more complex lesions that were excluded from the CAD I-IV studies.^16^^,^^27^, ^28^, ^29^ The study also has longer-term follow-up with outcomes up to 3 years. In all, the EMPOWER CAD study aims to directly address the underrepresentation of women in clinical trials through the collection of real-world clinical evidence for IVL in female patients with calcified CAD.

## Materials and methods

The EMPOWER CAD study is a postmarket, prospective, multicenter, single-arm observational study of the Shockwave Coronary IVL system for the treatment of women with calcified coronary arteries (Central Illustration). Details on the design and usage of the Shockwave Medical Coronary IVL system have been previously published.^25^ The study timeline will incorporate the transition from the Shockwave Coronary IVL C2 to the C2+ system with the use of either catheter allowed. Written informed consent is obtained before any study-specific requirements. All sites are required to follow local legal and regulatory requirements for ethics committee and institutional review board approvals. The Cardiovascular Research Foundation serves as the independent clinical events committee, data safety monitoring committee, and angiographic/optical coherence tomography (OCT) core laboratory. The study is being conducted in accordance with the guidelines of the Declaration of Helsinki and Good Clinical Practices.

**Central Illustration:**
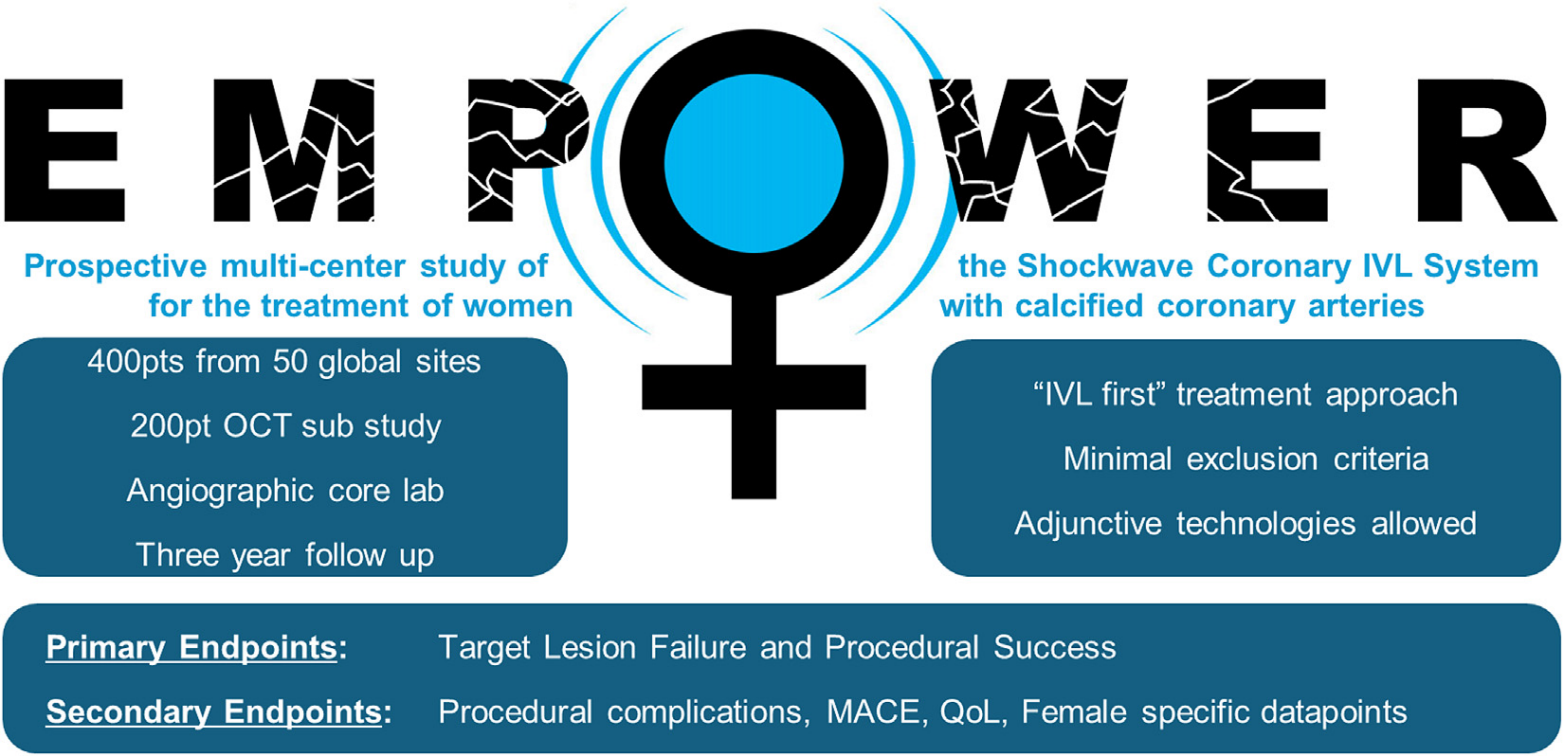
**EMPOWER CAD study overview.** IVL, intravascular lithotripsy; MACE, major averse cardiovascular events; OCT, optical coherence tomography; pts, patients; QoL, quality of life.

### Study cohort

The study will enroll 400 patients from up to 50 global sites. Female patients referred for PCI with coronary IVL and stenting per standard of care are eligible for the study provided they meet the inclusion and exclusion criteria (Table 1). Unlike the Disrupt CAD III and IV trials, which required that the target lesion be severely calcified with lesion lengths of <40.0 mm,^16^^,^^29^ the EMPOWER CAD study allows for longer lesions as well as those with moderate lesion calcification. The study will also allow for patients with multiple lesions, target lesions in ostial locations, unprotected left main lesions, or nonstented lesions previously treated with angioplasty.^16^^,^^29^ Patients presenting with in-stent restenosis as the target lesion will be excluded. Overall, exclusion criteria have been kept to a minimum to best assess the real-world use of coronary IVL.

**Table 1:** Inclusion and exclusion criteria for EMPOWER CAD study.

**Inclusion criteria**
Female patients (female sex assigned at birth) referred for percutaneous coronary intervention (PCI) with coronary intravascular lithotripsy (IVL) and stenting per standard of care will be assessed. Subjects are required to meet all of the following inclusion criteria in order to be enrolled in the clinical study. • The subject is a nonpregnant female aged 18 years or older • The subject meets indications for PCI and stent • The subject is sceduled to undergo PCI with coronary IVL and stenting per standard of care for nonstented lesion • The subject is willing to comply with protocol-specified follow-up evaluations • The subject, or legally authorized representative, has been informed of the nature of the study, agrees to its provisions, and has provided written informed consent, approved by the appropriate institutional review board or ethics committee
**Exclusion criteria**
Subjects who meet any of the following exclusion criteria may not be enrolled in the study: • Subjects with known mental or physical illness or known history of substance abuse that may cause noncompliance with the protocol, confound the data interpretation, or is associated with a life expectancy of less than 1 year • Subjects presenting with cardiogenic shock at the time of the index procedure • Serious angiographic complication in the target vessel before treatment with coronary IVL including-severe dissection (type D to F), perforation, abrupt closure, persistent slow flow, or persistent no reflow • Subject unable to tolerate anticoagulation/antiplatelet therapy per guidelines • Subject is enrolled in any study of an investigational device or drug that may interfere with study results

### Primary, secondary end points, and follow-up schedule

The primary safety end point is target lesion failure (TLF) at 30 days, defined as a composite of cardiac death, MI attributable to target vessel, or ischemia-driven target lesion revascularization. MI is defined using the Society for Cardiovascular Angiography & Interventions (SCAI) definition for periprocedural MI and the Fourth Universal Definition for spontaneous MI beyond discharge.^30^^,^^31^ This is in accordance with expert consensus on the definition of clinically relevant MIs following coronary revascularization. The primary effectiveness end point is procedural success, defined as stent delivery with a residual in-stent stenosis ≤30% in all target lesions and without in-hospital TLF. Residual in-stent stenosis and in-hospital TLF are assessed by the core laboratory and clinical events committee adjudicated, respectively.

Secondary end points are listed in Table 2. Composite end points of residual stenosis of ≤30% in all target lesions without serious angiographic complications or with residual stenosis thresholds of <50% will be examined. Serious angiographic complications are defined as severe dissection (type D to F), perforation, abrupt closure, and persistent slow flow or persistent no reflow. Additional secondary end points include MACE, angina symptoms as a change from baseline assessed by Seattle Angina Questionnaire (SAQ-7), and quality of life assessed by European Quality of Life 5 Dimension 5 Level (EQ-5D-5L) and Generalized Anxiety Disorder (GAD)-7 questionnaires. MACE is defined as a composite of cardiac-related death, MI, and target vessel revascularization at 30 days, 1, 2, and 3 years.

**Table 2:** Secondary end points in EMPOWER CAD study.

Secondary end points • Angiographic success defined as stent delivery with ≤30% residual stenosis and without serious angiographic complications. • Procedural success defined as stent delivery with a residual stenosis <50% in all target lesions (core laboratory assessed) and without in-hospital TLF. • Angiographic success defined as stent delivery with <50% residual stenosis and without serious angiographic complications. • Serious angiographic complications defined as severe dissection (type D to F), perforation, abrupt closure, and persistent slow flow or persistent no reflow. • TLF at 1, 2, and 3 y. • Major adverse cardiac events (MACE) defined as a composite of cardiac death, myocardial infarction (per SCAI definition for periprocedural MI; per Fourth Universal Definition for spontaneous MI beyond discharge), and target vessel revascularization (TVR) at 30 days and 1, 2 and 3 y. • At each period: all-cause death, cardiac-related death, MI, TV-MI, procedural and nonprocedural MI, ID-TVR, ID-TLR, ID-non-TLR, ID-non-TVR, all revascularizations (ID and non-ID), and stent thrombosis (Academic Research Consortium definite, probable, definite or probable). • MI rates and all composite end points (TLF, MACE) will also be reported using the Fourth Universal definition for periprocedural and spontaneous MI at all time points. • Angina symptoms assessed by Seattle Angina Questionnaire (SAQ-7) as a change from baseline (at each period). • Quality of life assessed by EQ-5D-5L as a change from baseline (at each period). • Quality of life assessed by Generalized Anxiety Disorder Questionnaire (GAD-7) as a change from baseline (at each period).

CAD, coronary artery disease; ID, ischemia driven; MI, myocardial infarction; TLF, target lesion failure.

Clinical follow-up is scheduled to occur at 30 days and 1, 2, and 3 years. Female-specific data points, SAQ-7, EQ-5D-5L, GAD-7, antiplatelet/anticoagulation medications, and adverse events will also be assessed at these visits. Female-specific data points include, but are not limited to, pregnancy history, menopausal status, hormone replacement therapy, past and current use of hormonal contraceptives, hysterectomy, and oophorectomy status.

### Statistical methods

Descriptive statistics will be conducted at prespecified time points of 30 days and 1, 2, and 3 years. For categorical variables, comparisons will use a χ^2^ test, Fisher exact test, or McNemar χ^2^. Exact CIs will be generated for estimates of proportions. For continuous variables, within-patient changes will be analyzed parametrically using the paired *t* test or nonparametrically using the sign-rank test. Statistical analyses will be performed using SAS version 9.4 or higher (SAS Institute).

### Sample size determination

The calculation of the EMPOWER CAD study sample size is based on a procedural success estimate of 90.0% from the Disrupt CAD III female subgroup results.^32^ With a desired precision of ±3.1% and an α of 0.05 (95% CI), a sample size of 360 provides approximately 80% power. To account for a potential lost-to-follow-up rate of 10%, total enrollment is set at 400 patients.

### Treatment algorithm

**Figure 1:**
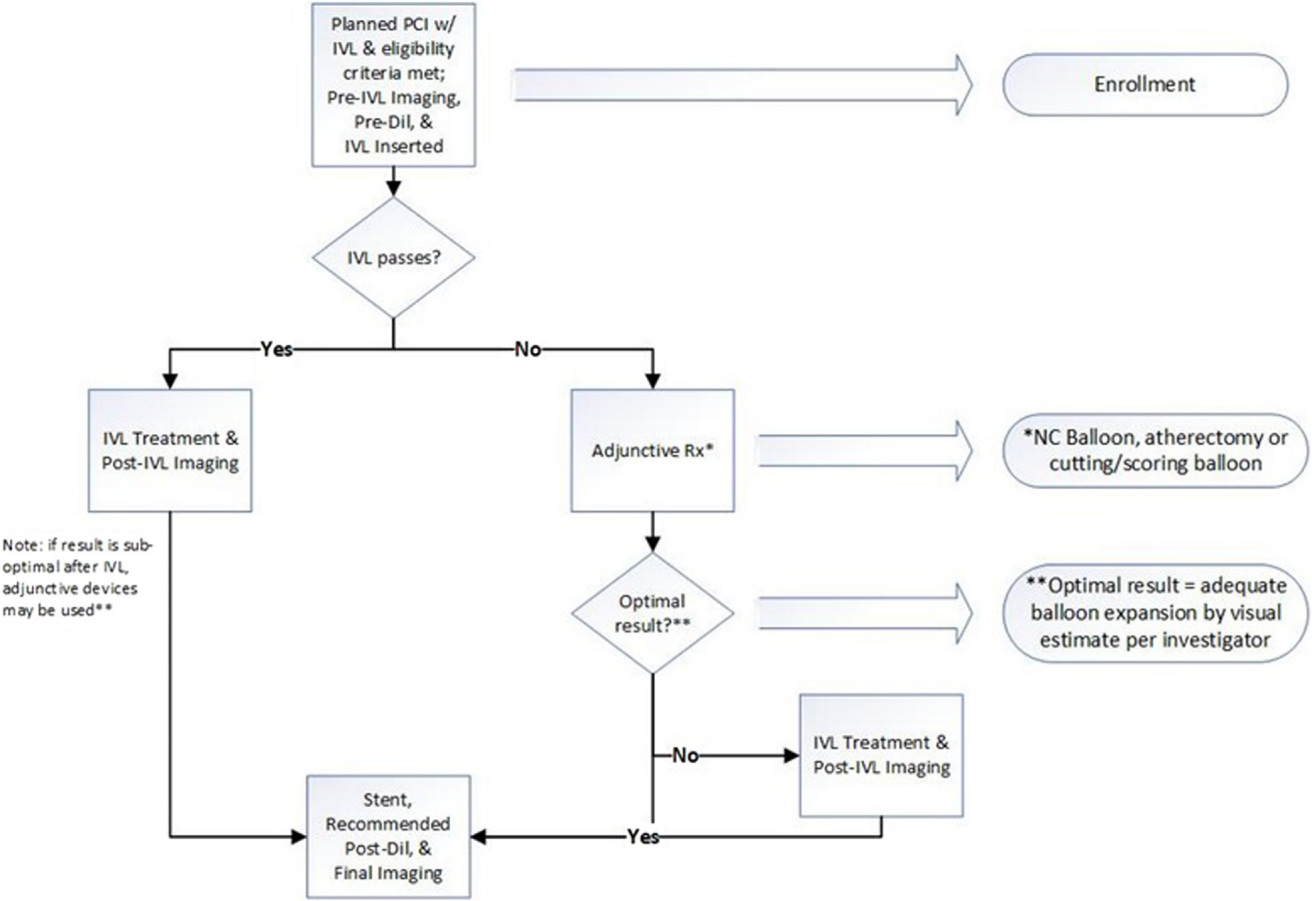
**EMPOWER CAD study treatment algorithm.** Dil, dilation; IVL, intravascular lithotripsy; NC, noncompliant; PCI, percutaneous coronary intervention.

In the EMPOWER CAD study, an “IVL first” approach is recommended (Figure 1, Appendix A). Briefly, the protocol strongly recommends treating nontarget lesions first if a patient has multiple lesions. The patient is enrolled at the time when the decision to use IVL first is confirmed and the device is opened. If the investigator can pass a guide wire but is unable to pass the IVL catheter across the target lesion, an adjunctive tool (balloon, atherectomy, cutting/scoring balloon) may be used before reattempting passage of the IVL catheter. The lesion is then to be treated per IVL instructions for use. If the IVL does not pass despite adjunctive treatment, this will be captured, and the patient will remain in the study. If the adjunctive treatment sufficiently modifies the lesion, the operator can elect not to proceed to use IVL, but again this will be captured, and the patient will remain in the study.

If residual stenosis is suboptimal following the IVL procedure, adjunctive devices (balloon, atherectomy, cutting/scoring balloon) may be used to dilate the lesion before stenting. The use of adjunctive devices, including the order in which they are used, will be documented. With multiple calcium modification technologies available, several studies have presented algorithms in an effort to optimize treatment strategy, assessment of successful calcium modification, and stent optimization.^33^, ^34^, ^35^ While most studies compared the safety and effectiveness of one device against another, further information is needed on the adjunctive use of calcium modification technologies, as this likely is more representative of real-world practice.^36^^,^^37^

### OCT substudy and other subgroup analyses

Up to 200 patients who consent and have an adequate set of OCT images captured will be enrolled in the optional OCT substudy at preselected qualified institutions where OCT is routinely performed as standard of care. With the all-comers design of the trial, we anticipate a more comorbid and complex study cohort than in previous IVL trials, and thus, there is some uncertainty regarding the proportion of subjects who will be suitable for OCT. For these patients, OCT images will be collected at baseline, immediately post-IVL, and end of procedure (poststent/postdilation) and assessed by the core laboratory. Advanced imaging allows physicians to choose an appropriate calcium modification strategy and to achieve optimal PCI results.^33^^,^^35^^,^^38^ The importance of intravascular ultrasound or OCT for quantifying calcification and guiding PCI has been recognized in the 2021 ACC/AHA/SCAI Coronary Artery Revascularization guidelines^5^ because of the high sensitivity and specificity of these imaging techniques.^39^^,^^40^ The OCT substudy will allow for quantification of the severity of calcification, assessment of the prevalence of different calcium morphologies, and assessment of IVL modification and stent outcomes.

The primary safety and effectiveness end points will be compared for other subgroup analyses using a logistic regression model including an intercept term and fixed effect for the subgroup with a corresponding 95% CI and *P* value. An example subanalysis would be comparing those older and younger than 75 years, as PCI is becoming more common in elderly patients who typically have higher calcification.^41^ With the documentation of the order in which adjunctive devices were used, a subanalysis on different treatment strategies to optimize calcium modification would be beneficial.^33^, ^34^, ^35^ Finally, as severe calcification is associated with adverse events such as dissection, restenosis, and target vessel revascularization,^17^, ^18^, ^19^ comparisons of patients grouped by calcium severity are possible with the EMPOWER CAD data set.

### Ethics statement

This research will be carried out in accordance with ethical guidelines. Ethics committee approval was obtained, and all subjects will provide written informed consent.

### Women in interventional cardiology

Elevating women is an important and unique element of the EMPOWER CAD study. As described earlier, women are consistently underrepresented in previous clinical trials,^6^, ^7^, ^8^, ^9^ and the lack of data-driven sex-specific treatment algorithms may be leading to the increased risk of mortality and adverse events for women.^9^^,^^11^^,^^14^ In addition, while women make up 50% of medical school graduates, only 10% of practicing cardiologists and only 3% to 5% of interventional cardiologists are women.^42^, ^43^, ^44^ There is a need for more career advancement opportunities^45^ as well as programs like SCAI Women in Innovations (WIN) to further professional development and networking for female operators.^44^ In the EMPOWER CAD study, female physicians were nominated to be the principal investigators as recognition of their contributions to the field and to provide an opportunity to increase representation in interventional cardiology research.

## Conclusions

The EMPOWER CAD study is a rigorously designed study that assesses the real-world use of the Shockwave Coronary IVL system for the treatment of women with calcified coronary arteries. The study design allows for female all-comer patients excluded from previous coronary IVL studies and will provide outcomes up to 3 years. The treatment algorithm assesses adjunctive use of IVL with other calcium modification technologies as this likely is more representative of real-world practice. Overall, the EMPOWER CAD study addresses a critical evidence gap by studying a currently underrepresented population in CAD PCI clinical trials and provides much-needed guidance for the use of calcium modifying tools in women.
