# Supplementary material for: Equity in Modifying Plaque of Women With Undertreated Calcified Coronary Artery Disease: Design and Rationale of EMPOWER CAD study
Source: J Soc Cardiovasc Angiogr Interv. 2024 Oct 28;3(11):102289. doi: 10.1016/j.jscai.2024.102289 (PMC11624350; doi:10.1016/j.jscai.2024.102289)
Supplement: Appendix A [file mmc1.docx]

**APPENDIX A**

IVL Treatment Algorithm

A full description of the IVL procedure is detailed in the Instructions for Use (IFU) including appropriate balloon sizing. Note that there are specific IFUs and labelling provided for the United States (US) and for Outside of the US (OUS). An appropriately-sized IVL catheter should be selected per the IFU.

The recommended procedural steps outlined below represent an “IVL first” approach; adjunctive devices may be used if there are challenges after first attempting to cross with the IVL catheter. It is strongly recommended that operators follow the treatment algorithm. Any departures from this algorithm will not be considered protocol deviations; information on all devices used in the procedure (including the order in which they were used) will be captured. Angiographic images captured during the procedure will be sent to the core lab for analysis.

- If multiple lesions are to be treated, it is recommended that non-target lesions be preferentially treated first.
- If a serious angiographic complication occurs in a target or non-target vessel prior to insertion of the IVL catheter, the subject should be treated per standard of care; however, they no longer meet eligibility criteria and will not be enrolled in the study.
- If the Investigator is able to pass a guidewire but is unable to pass the IVL catheter across the target lesion, an adjunctive tool (balloon, atherectomy, cutting/scoring balloon) may be used prior to re-insertion of the IVL Catheter.
- If an optimal result is achieved following treatment with adjunctive device, study team may proceed per standard of care
- If optimal result is not achieved following treatment with adjunctive device, the IVL catheter may be re-inserted and the lesion will then be treated per the IFU with the IVL Catheter.

*Note: Pre-dilatation may be performed using standard techniques based on physician discretion. The subject is considered enrolled once the IVL Catheter has been inserted* *into*

*the access artery.*

- Once the IVL catheter is placed in the target lesion area, the balloon should be inflated to 4 atm and IVL treatment delivered for the pre-programmed time of 10 seconds to deliver 10 pulses.

*Note: The IVL Generator is programmed to force a minimum pause time of 10 seconds following every 10 pulses delivered.*

- Following IVL, inflate the balloon to the reference size using the balloon compliance chart (refer to IFU) and record lesion response on fluoroscopy.
- Deflate the balloon to re-establish blood flow.
- Repeat prior steps for additional treatment cycles until the lesion has been sufficiently dilated or the catheter is repositioned.
- If additional lesion area needs to be treated, follow the treatment steps identified above and per the IFU to ensure appropriate overlap between segments.

*Note: The maximum number of pulses to treat a single arterial segment is 80 pulses and therefore 160 pulses in an overlap segment. Additional catheters may be used when necessary.*

- The residual stenosis will be assessed by the physician following the IVL procedure. The IVL procedure is considered successful when the residual stenosis allows for adequate balloon expansion by visual estimate, as determined by the Investigator prior to stent placement. Consider additional IVL pulses if needed to optimize residual stenosis.
- If the residual stenosis is sub-optimal following IVL, a balloon or other adjunctive device (atherectomy, cutting/scoring balloon) must be used to dilate the lesion prior to stenting. This information will be recorded in the case report form.
- Post-IVL angiography will be performed after IVL pulses have been delivered and prior to stent delivery.
- The stent will then be delivered using a standard approach.
- Following stent implantation, post-dilatation with a non-compliant balloon with inflation pressure ≥16 atm is strongly recommended.
- Following delivery of the coronary stent and post-dilatation, angiography will be performed to determine the final residual stenosis for assessment of the primary effectiveness endpoint.
